# Neighborhood Environmental Interventions and Opioid Overdose Rates

**DOI:** 10.1001/jamanetworkopen.2026.26634

**Published:** 2026-07-31

**Authors:** Eugenia C. South, Emily Seeburger, Abby Dolan, Greg Ridgeway, Shoshana Aronowitz, Charles C. Branas, Jeanmarie Perrone, John MacDonald, Douglas Wiebe, David S. Mandell, Kehinde Oyekanmi, Nicole Thomas, Zachary F. Meisel

**Affiliations:** 1 Penn Medicine Center for Health Justice, University of Pennsylvania Perelman School of Medicine, Philadelphia; 2 Center for Emergency Care Policy and Research, Department of Emergency Medicine, University of Pennsylvania Perelman School of Medicine, Philadelphia; 3 Department of Criminology and Sociology, University of Pennsylvania, Philadelphia; 4 Department of Statistics and Data Science, University of Pennsylvania, Philadelphia; 5 Department of Family and Community Health, University of Pennsylvania School of Nursing, Philadelphia; 6 Department of Epidemiology, Mailman School of Public Health, Columbia University, New York, New York; 7 Center for Addiction Medicine and Policy, University of Pennsylvania Perelman School of Medicine, Philadelphia; 8 Department of Epidemiology, School of Public Health, University of Michigan, Ann Arbor; 9 Center for Mental Health, Department of Psychiatry, University of Pennsylvania Perelman School of Medicine, Philadelphia; 10 Penn Injury Science Center, University of Pennsylvania Perelman School of Medicine, Philadelphia; 11 Department of Emergency Medicine, University of Pennsylvania Perelman School of Medicine, Philadelphia

## Abstract

**Importance:**

Previous research has shown neighborhood environmental interventions can improve health and safety for residents. It is unknown whether these interventions impact opioid overdose outcomes.

**Objective:**

To evaluate the association of neighborhood environmental interventions with opioid overdose rates.

**Design, Setting, and Participants:**

This quasi-experimental nonrandomized trial used a continuous treatment difference-in-differences design and was conducted in a single neighborhood of Philadelphia, Pennsylvania, between January 1, 2019, and December 31, 2021. The quasi-experimental design used nonrandomized comparator groups to evaluate the associations of neighborhood environmental interventions with opioid overdose rates since randomization was not possible. Data analysis took place between May 2022 and February 2025.

**Interventions:**

Community trash pickups, vacant lot cleanups, and abandoned house remediation.

**Main Outcomes and Measures:**

Outcomes of interest were nonfatal and fatal opioid overdoses.

**Results:**

Of 1667 individual blocks (median household income, $37 821) included in the study geography, 622 blocks (37.3%) received at least 1 intervention during the study period. Among 59 590 residents in the study area, there were 1179 Asian residents (2.0%), 9951 Black residents (16.7%), 23 261 White residents (39.0%), 5016 residents (8.4%) who identified as 2 or more races, and 19 903 residents (33.4%) who identified as another race; 30 286 residents (50.8%) were Hispanic or Latinx. A total of 763 community trash pickups, 483 abandoned house remediations, and 855 vacant lot cleanups were included in analysis. In aggregate, the interventions were associated with a significant reduction in fatal opioid overdoses (change, −6.6%; 95% CI, −10.5% to −2.4%), with no measurable association for nonfatal opioid overdoses (change, −0.0%; 95% CI, −3.4% to 3.5%). Abandoned house remediation was associated with a significant reduction in both fatal (change, −17.6%; 95% CI, −26.2% to −7.9%) and nonfatal (change, −11.5%; 95% CI, −19.0% to −3.3%) opioid overdoses. Community trash pickup was not associated with rates of fatal or nonfatal overdoses. Vacant lot cleanup was not associated with rates of fatal overdoses but was associated with a significant increase in nonfatal overdoses (change, 11.7%; 95% CI, 4.0% to 20.0%). No significant displacement associations were found in other locations.

**Conclusions and Relevance:**

In this study of the association of neighborhood environmental interventions with opioid overdose rates, interventions analyzed in aggregate were associated with reduced fatal opioid overdoses outcomes, with no association with nonfatal outcomes. Abandoned house remediation was significantly associated with reduced fatal and nonfatal opioid overdose rates and should be considered along with other essential interventions that provide treatment and harm reduction to individuals with substance use disorder.

## Key Points

**Question:**

Are neighborhood environmental interventions associated with reductions in fatal and nonfatal opioid overdoses?

**Findings:**

In this nonrandomized trial including 1667 blocks of a single neighborhood of Philadelphia, Pennsylvania, aggregate interventions were associated with a significant reduction in fatal opioid overdose, with no association for nonfatal opioid overdose. Abandoned house remediation was associated with a significant reduction in fatal and nonfatal opioid overdoses, community trash pickup was not associated with fatal or nonfatal overdoses, and vacant lot cleanup was not associated with fatal overdoses but was associated with a significant increase in nonfatal overdoses.

**Meaning:**

These findings suggest that abandoned house remediation should be considered a viable community-level intervention for opioid overdose.

## Teaser

This nonrandomized clinical trial evaluates the association of neighborhood environmental interventions with opioid overdose rates in Philadelphia, Pennsylvania.

## Introduction

The opioid crisis continues in the US, and Philadelphia, Pennsylvania—with a death rate more than twice the national average—is considered a bellwether for the national crisis.^1,2,3^ Overdoses are spread throughout the city, with the highest concentration in the study neighborhood.

A neighborhood’s physical conditions affect residents’ health in myriad ways, including substance use behaviors and outcomes, such as access to drugs, initiation of use, severity of addiction, and risk of overdose and death.^4,5,6,7,8,9,10,11,12,13,14,15,16,17,18,19,20,21^ Cities with higher prevalence of dilapidated environmental conditions, including abandoned buildings and trash, have higher rates of injection drug use.^22^ In Philadelphia, zip codes with more vacant housing and lower housing quality have been associated with increased overdose mortality.^23^

Several mechanisms link substance use to dilapidated neighborhood conditions. Residents cite vacant and trash-filled spaces as decreasing social order, fracturing ties among neighbors, increasing crime and safety-related stress, and undermining mental health, all of which are risk factors for substance use and overdose.^24^ People living in neighborhoods with high levels of blight, for example, are at higher risk for psychological distress and heavy drinking.^19^ Additionally, vacant lots and abandoned buildings may provide opportunities for selling, buying, and using opioids.^25^

Prior research demonstrates neighborhood environmental interventions can improve health and safety for residents. Two randomized trials of vacant lot greening and abandoned house remediation in Philadelphia demonstrated reductions in violent crime and improvements in social cohesion and mental health.^25,26,27^ Structural repairs to occupied homes in historically disinvested neighborhoods have also been associated with decreases in crime.^28^ Despite links between neighborhood conditions and opioid overdose outcomes and evidence suggesting environmental interventions are associated with improved health and safety, no published studies have evaluated if such interventions can impact opioid overdose outcomes, to our knowledge.

To address this gap, we evaluated several City of Philadelphia neighborhood environmental interventions—vacant lot cleanup, abandoned house remediation, and community trash pickups—in the neighborhood with the highest rate of fatal overdoses. We hypothesized these interventions would be associated with reductions in fatal and nonfatal opioid overdose compared with areas in the same neighborhood without these interventions.

## Methods

This quasi-experimental^29^ nonrandomized clinical trial used a difference-in-differences design to analyze the association of City of Philadelphia neighborhood environmental interventions with opioid overdose rates from January 1, 2019, to December 31, 2021, in a single neighborhood. The quasi-experimental design was chosen because using nonrandomized comparator groups allowed us to evaluate the impact of an intervention since randomization was not possible.^29^ The institutional review boards at the University of Pennsylvania and the City of Philadelphia approved the study. Informed consent was waived by the University of Pennsylvania institutional review board due to use of administrative data only. We followed the Transparent Reporting of Evaluations With Nonrandomized Designs (TREND) reporting guideline. The trial protocol and statistical analysis plan are provided in Supplement 1.

### Neighborhood Environmental Interventions

In 2018, Philadelphia instituted Opioid Emergency Executive Order No. 3-18, spurring 35 city agencies to form the Opioid Emergency Response Group.^30^ The resulting Philadelphia Resilience Project was a multiyear effort to address neighborhood-level overdose prevention in the neighborhood with the highest rate of fatal overdoses in the city. The Resilience Project included direct services to people with substance use disorder (SUD) and several neighborhood environmental interventions addressing dilapidated conditions. These interventions included community trash pickups, vacant lot cleanup, and abandoned house remediation.

Community trash pickups generally targeted a single block or block cluster and were initiated by community members or other local organizations through the city’s Community Life Improvement Project (CLIP) Community Partnership Program, which loans equipment and supplies for cleanup events (eg, rakes, brooms) and coordinates trash pickup afterwards. CLIP also conducted 1-time vacant lot cleanups, removing trash and mowing grass on vacant lots in violation of city ordinance. Vacant lots were identified either through a 311 complaint or when a city inspector investigated a reported vacant lot and identified other nearby vacant lots in violation. No maintenance was included in this intervention.

Abandoned houses were also identified through complaints to the city or city inspector. Abandoned house remediation initially included 2 separate interventions. The first included CLIP sealing doors and windows of abandoned houses with plywood to prevent entrance. The second intervention involved painting the plywood to appear as if doors and windows were installed.

The city of Philadelphia provided community trash pickup and painted doors and windows intervention data. Vacant lot cleanup and abandoned house remediation data were accessed through the city’s open data library, OpenDataPhilly.

### Outcome Measures

To determine fatal overdoses, we used data from Philadelphia’s Medical Examiner’s Office. We included all deaths the Medical Examiner’s Office attributed to an accidental overdose involving a prescription, synthetic, or other opioid that occurred in our study geography during study dates. To determine nonfatal overdoses, we used data from the Philadelphia Fire Department emergency medical service (EMS) runs. EMS runs were included if they occurred in our study geography during study dates and if the electronic patient care report indicated the patient had experienced an opioid overdose (determined through an on-scene assessment, information from the dispatcher, or information from bystanders on scene) or the paramedic indicated they administered naloxone on scene. We focused on opioid overdose to align with the goals of the Resilience Project and because most overdoses in Philadelphia involve opioids. More detail regarding nonfatal overdose determination is provided in the eMethods in Supplement 2, and sensitivity analyses, including acute opioid intoxication and nonopioid intoxication, are presented in eTable 1 and eTable 2 in Supplement 2.

### Dataset Creation

Vacant lot cleanup and abandoned house remediation data included latitude and longitude coordinates. Addresses were geocoded to obtain coordinates for community trash pickups. We joined fatal overdoses, nonfatal overdoses, and environmental interventions to their nearest city block. City blocks were derived from the city’s Street Centerline shapefile. Merging all data produced a database accounting for all outcomes and interventions by block in the study area. Next, using intervention, patient care report, and death dates, we counted the week and cumulative weekly number of each intervention, the sum of all interventions, and the number of nonfatal and fatal overdoses.

### Statistical Analysis

To describe the neighborhood-level demographics, we obtained data from the 2021 US Census Bureau American Community Survey 5-year estimates. Race and ethnicity of patients experiencing overdose were determined by the responding paramedic and categorized as African American or Black, American Indian or Alaska Native, Asian, Native Hawaiian or Other Pacific Islander, White, 2 or more races, and another race, with ethnicity classified as Hispanic or Latinx or not Hispanic or Latinx. We include race and ethnicity information to describe the demographics of opioid overdose during our study period in Philadelphia.

The unit of analysis was the city block, similar to prior work evaluating neighborhood environmental interventions, and because neighborhood social life is often organized around blocks.^28^ Blocks on which there was no intervention during the study period served as the control comparison for our analysis. Blocks on which there were 13 or more interventions, representing blocks at and above the 90th decile, were removed as outliers.

Of the interventions initially selected for study, the painted doors and windows intervention was halted in response to the COVID-19 pandemic on March 16, 2020, and never resumed. Consequently, this intervention had a limited amount of data and was dropped from our analysis due to a lack of statistical power. Final analysis included community trash pickups, vacant lot cleanup, and abandoned house clean and seal, which we refer to as abandoned house remediation.

We used a continuous-treatment difference-in-differences conditional quasi-Poisson cumulative dose panel model to estimate the associations between neighborhood environmental interventions on fatal and nonfatal overdoses. The model incorporated block-specific outcomes and smooth shared temporal trends to account for time-invariant differences across blocks and secular changes in overdose rates over time. We chose this design to mitigate various threats to validity, including neighborhoodwide historical trends and regression to the mean. Coefficients were converted to percentage changes in fatal and nonfatal overdose rates using baseline data of mean monthly fatal and nonfatal overdoses in the neighborhood. This method of interpretation better contextualizes the data and aligns with the goal of the Resilience Project to reduce overdose at the neighborhood level.

Formally, we fit the model log*E*(count*_tj_*) = β_1_CommunityServ*_tj_* + β_2_VacantLot*_tj_* + β_3_SealClean*_tj_* + *α_j_* + *ns*_12_(*t*), where *count_tj_* is the outcome (number of fatal or nonfatal overdoses) on block *j* in week *t* of the study period. CommunityServ*_tj_* is the cumulative number of community service projects on block *j* by week *t*. Similarly, VacantLot*_tj_* and SealClean*_tj_* are the cumulative counts of vacant lot cleanups and abandoned house remediations, respectively. We used *α_j_* as a block–fixed effect to control for time-stable, unmeasured differences between blocks. Estimation proceeded using the conditional Poisson likelihood, which conditions out these block-specific effects rather than estimating them directly. To account for shared time trends, we include a natural cubic spline with 12 degrees of freedom, denoted *ns*_12_(*t*). We also fit a separate any intervention model that collapsed the environmental interventions into 1 regression term, β*_Any_*(CommunityServ*_tj_* + VacantLot*_tj_* + SealClean*_tj_*).

Estimated associations were identified from within-block changes in cumulative intervention exposure over time relative to concurrent temporal trends among never-treated blocks. This specification extends the conventional difference-in-differences framework to our continuous, cumulative treatment setting, given there is no clear pre-post time period and interventions in our study are measured by counting the total number of activities conducted on the block cumulatively over time.

We conducted a permutation test to check the validity of these results by assessing the statistical significance of the association between independent variables and the dependent variable while making minimal assumptions about the underlying data distribution.^31^ Permutation tests do not rely on traditional parametric assumptions, such as normality. Instead, we randomly shuffled the values of the dependent variable across observations multiple times, refitting the regression model each time, and comparing the observed coefficients to a distribution of coefficients generated from the permutations. This provides a different way to determine the probability that the observed associations between variables is due to chance (eMethods in Supplement 2).

We also tested whether the weekly count of each intervention could be projected by the preceding week’s count of fatal and nonfatal overdoses to ensure our model did not violate the assumption that the timing of interventions is not impacted by the outcome. Formally, we fit the model log*E*(Environments*_tj_*) = *β*_1_Fatal*_t_*_−1_*_j_* + *β*_2_*Nonfatal_t_*_−1_*_j_* + *α_j_* + *ns*_12_(*t*).

Hypothesis tests were 2-sided with *P* < .05 indicating statistical significance. All analyses were performed with R software version 4.3.1 (R Project for Statistical Computing) with generalized nonlinear models.^32^ Finally, we conducted a sensitivity analysis based on the COVID-19 pandemic, which occurred in the middle of the study period and had a known association with opioid overdose rates (eMethods and eTable 3 in Supplement 2). Data analysis took place between May 2022 and February 2025.

## Results

### Neighborhood, Intervention, and Individual Characteristics

In our study area, the median household income was $37 821. Among 59 590 residents, there were 1179 Asian residents (2.0%), 9951 Black residents (16.7%) , 23 261 White residents (39.0%), 5016 residents (8.4%) who identified as 2 or more races, and 19 903 residents (33.4%) who identified as another race; 30 286 residents (50.8%) were Hispanic or Latinx.^33^ The unemployment rate was 6.7%. We identified 1667 individual blocks in our study geography, of which 622 (37.3%) received at least 1 intervention over the study period. On blocks that received an intervention, there was a mean (SD) of 4 (3) interventions per block. A total of 763 community trash pickups, 483 abandoned house remediations, and 855 vacant lot cleanups were included in our analysis (Table 1 and Figure).

**Table 1.** Neighborhood, Intervention, and Individual Demographic Characteristics

Item	No. (%)
**Neighborhood-level US Census demographics**	
Sex	
Female	31 634 (53.1)
Male	27 956 (46.9)
Race	
African American or Black	9951 (16.7)
American Indian or Alaska Native	267 (<1.0)
Asian	1179 (2.0)
Native Hawaiian or other Pacific Islander	13 (<1.0)
White	23 261 (39.0)
≥2 Races	5016 (8.4)
Another race	19 903 (33.4)
Ethnicity	
Hispanic or Latinx	30 286 (50.8)
Not Hispanic or Latinx	29 304 (49.2)
Household income, median (IQR), $	37 821
Unemployment rate, %	6.70
**Intervention characteristics**	
City blocks in study area, No.	1667
Blocks receiving intervention, No.	622 (37.3)
Neighborhood environment interventions	
Overall, No.	2101
Abandoned house remediation	483 (23.0)
Community trash pickups	763 (36.3)
Vacant lot cleanups	855 (40.7)

**Figure.**
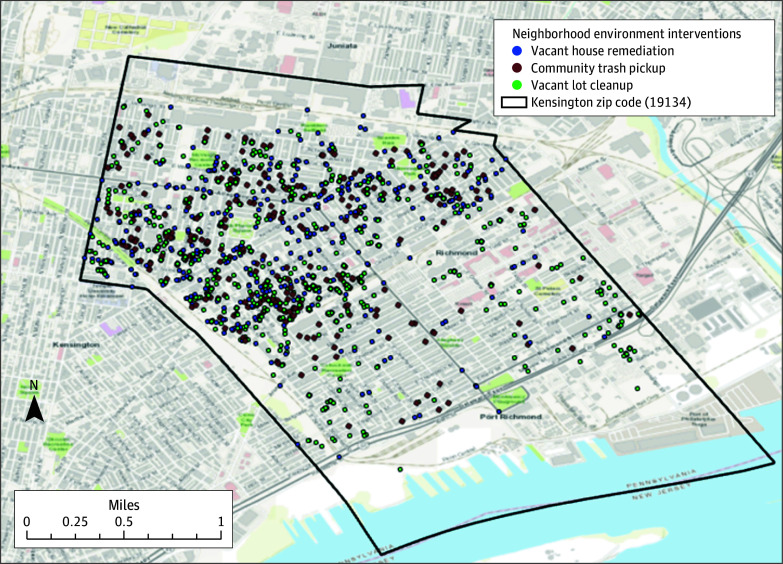
Map of All Neighborhood Environment Interventions in Study Area

During the study period, 3056 fatal opioid overdoses occurred across all 93 Philadelphia zip codes, with 310 fatal overdoses (9.9%) occurring on city blocks included in our study. Individuals who died from opioid overdose in our study included 250 (80.5%) males, 224 White individuals (72.5%), and 82 Hispanic or Latino individuals (26.5%), with a mean (SD) age of 44 (12) years. A total of 10 529 EMS-identified nonfatal opioid overdoses occurred across the city during the study period, with 849 nonfatal opioid overdoses (8.1%) occurring on city blocks included in our study. Individuals who experienced a nonfatal overdose in our study included 547 (64.4%) males, 348 White individuals (41.0%), and 196 Hispanic or Latinx individuals (23.1%), with a mean (SD) age of 39 (14) years (Table 2).

**Table 2.** Demographics for Individuals Experiencing Opioid Overdose Included in Study

Characteristic	Individuals, No. (%)	
	Fatal	Nonfatal
Total opioid overdoses in Philadelphia, No.	3056	10 529
Total opioid overdoses in study	310 (10.1)	849 (8.1)
Sex		
Female	60 (19.4)	188 (22.1)
Male	250 (80.7)	547 (64.4)
Missing	0	114 (13.4)
Race		
African American or Black	80 (25.9)	150 (17.7)
Asian	3 (1.0)	0 (0.0)
White	224 (72.5)	348 (41.0)
Other	2 (1.0)	41 (4.8)
Missing	1 (<1.0)	114 (13.4)
Ethnicity		
Hispanic or Latinx	82 (26.5)	196 (23.1)
Missing	13 (4.2)	114 (13.4)
Mean (SD) age, y	44 (12.0)	39 (13.5)

### Fatal and Nonfatal Opioid Overdose Rates

The interventions were associated with a significant reduction in fatal opioid overdose (change, −6.6%; 95% CI, −10.5% to −2.4%), with no measurable association with nonfatal opioid overdose (change, −0%; 95% CI, −3.4% to 3.5%) on a given block (Table 3). Abandoned house remediation was associated with a significant reduction in both fatal (change, −17.6%; 95% CI, −26.2% to −7.9%) and nonfatal (change, −11.5%; 95% CI, −19.0% to −3.3%) opioid overdoses. Community trash pickup was not associated with fatal or nonfatal overdoses. Vacant lot cleanup was not associated with fatal overdose but was associated with a significant increase in nonfatal overdose (change, 11.7%; 95% CI, 4.0% to 20.0%) (Table 3). In our study geography, mean (SD) monthly nonfatal and fatal overdoses were 21 (11) and 15 (10), respectively, translating to no change in nonfatal overdoses and approximately 35 fewer fatal overdoses in total over the study period associated with the neighborhood environmental interventions. Our analysis assessing whether future environmental interventions were associated with overdose counts in the preceding week found no significant associations for any individual intervention type or for interventions in aggregate, for either fatal or nonfatal overdoses.

**Table 3.** Associations of Neighborhood Environment Interventions With Fatal and Nonfatal Opioid Overdoses in Study Area, Philadelphia, 2019-2021^a^

Outcome	Change, % (95% CI)			
	Any intervention^b^	Community trash cleanup	Abandoned house remediation	Vacant lot cleanup
Fatal overdoses	−6.55 (−10.51 to −2.42)^c^	1.32 (−5.10 to 8.18)	−17.56 (−26.20 to −7.91)^c^	−4.94 (−12.52 to 3.31)
Nonfatal overdoses	−0.02 (−3.41 to 3.49)	−1.03 (−5.46 to 3.60)	−11.49 (−19.02 to −3.26)^d^	11.69 (3.99 to 19.97)^d^

^a^ Over the course of 160 weeks, including 622 treated blocks and 1045 never-treated blocks.

^b^ Any intervention refers to the cumulative count of all intervention activities combined, regardless of intervention type.

^c^ *P* < .001 (vs no intervention).

^d^ *P* = .001 (vs no intervention).

### Displacement Analysis

One concern with neighborhood environmental interventions is that the outcomes of interest may simply be displaced to a different location. In prior work, vacant lot greening and abandoned house remediation were not associated with any meaningful spillover of crime to surrounding blocks.^27^ To evaluate for spillover, we took all blocks that received no intervention throughout the study period and joined them to their intersecting blocks that received any intervention, creating 1-to-1 or 1-to-many matches. While we left data on fatal and nonfatal overdoses by week for the nonintervention blocks unchanged, the weekly and cumulative intervention counts of the neighboring intervention blocks were then assigned to their nonintervention block matches. Then, we repeated our primary analysis, but with these substituted intervention counts. We hypothesized if there was displacement happening, we would see a statistically significant inverse change in fatal and nonfatal overdoses on nonintervention blocks. No significant displacement outcomes were noted (eTable 4 in Supplement 2).

## Discussion

In this quasi-experimental nonrandomized clinical trial of neighborhood interventions and opioid overdose outcomes, we found that a group of neighborhood environmental interventions in a section of Philadelphia with a high concentration of opioid overdose was associated with a modest but statistically significant reduction in fatal opioid overdoses. No association was found with nonfatal overdoses. Individually, abandoned house remediation was associated with fewer fatal and nonfatal overdoses, while 1-time vacant lot cleanups were associated with an increase in nonfatal overdoses.

Opioid use disorder is a complex medical condition requiring a range of multilevel interventions to reduce adverse outcomes. This study focused on community-level interventions in a community where public drug use intersects with poverty, historical disinvestment, and resultant dilapidated neighborhood conditions.

The finding that abandoned house remediation was associated with a reduction in fatal overdoses may be due to this intervention interrupting how dilapidated neighborhood spaces facilitate selling, buying, and using drugs. Abandoned houses are easily entered and can provide a semiprivate space in which to engage in these behaviors. Research in Philadelphia has demonstrated that drug sales occur outside of vacant properties and that vacant lots conceal drug use and provide escape routes during police raids.^25^ Abandoned house remediation may have prevented people from going alone into these spaces to use, perhaps pushing use to public spaces, meaning that if someone overdoses, others are around to administer naloxone or call for help. In neighborhoods like our study neighborhood with a high concentration of public drug use, safe consumption sites are needed to prevent overdose.

Vacant lot cleanups, in contrast, were associated with an increase in nonfatal opioid overdoses in this study. This differs from our prior work finding an association between lot cleanups and reductions in other unsafe activities, including firearm violence. In prior studies, vacant lots received biweekly trash cleanup (on the lot only, not the full block) and mowing, while in this study, lots did not receive maintenance after the initial cleanup and thus could fall quickly back into disrepair.^25^ It is also possible a cleanup may have facilitated increased drug use by providing a cleaner space to use drugs. These results do not mean vacant lots do not matter for health and safety outcomes. Instead, more sustainable interventions are likely needed (eg, regular maintenance) to see an impact on deeply entrenched problems, such as opioid overdose.

These mechanisms are supported by perspectives of members of our Community Advisory Board (CAB), individuals with firsthand experience of the study neighborhood and/or lived experience with SUD. Our study team formed the CAB to incorporate community member expertise to inform our research, and we met with them over the course of the study to help us contextualize our results. CAB members felt abandoned house remediation discourages people from using drugs in that area, specifically noting they do not tend to see people entering remediated houses and thus people tend to go elsewhere to use drugs, suggesting the interventions may not change whether a person uses drugs but may impact where they use them.

We did not see an association of community trash cleanups with overdose outcomes, which may be because trash likely quickly reaccumulates after 1-time pickups, thus negating the impact. Still, this does not mean there is no impact on the community. Our CAB members shared their perceptions that neighbors reported satisfaction with the cleanup efforts.

### Limitations

This study has several limitations. First, the study took place in an urban neighborhood; thus, the results should be generalized with caution. However, some evidence suggests the results may apply to the continuum of smaller urban to suburban neighborhoods that also have vacant land and abandoned structures. For example, a study evaluating opioid overdose trajectories in Passaic County, New Jersey, found that over a 5-year period, block groups with increasing rates of overdose had the highest amount of vacant land compared with block groups with low and stable or moderately increasing levels of overdose.^34^ Second, this study does not address the increasing rates of fatal and nonfatal overdoses among Black individuals both locally and nationally.^35^ From 2018 to 2022, fatal drug overdoses increased among Black residents of Philadelphia by 87% and Hispanic or Latinx residents by 43%, while decreasing for White residents by 12%. Due to the highly racially segregated nature of Philadelphia, our study took place in an area whose residents are primarily White and Hispanic or Latinx. How these interventions might impact communities with higher concentrations of Black individuals deserves further exploration.

Third, while our displacement analysis did not suggest displacement of overdoses to other areas in our study geography, we did not evaluate whether outcomes were displaced to neighborhoods surrounding our study geography. Fourth, the interventions studied here were not assigned at random and therefore are subject to various forms of confounding. Our quasi-experimental analytic design, allowing for comparisons before and after the interventions as well as across blocks that did and did not receive the intervention, gives weight to the consideration that the associations are less likely due to secular or other factors external to the interventions of interest.

## Conclusions

This nonrandomized clinical trial found that, in aggregate, neighborhood environmental interventions were associated with reduced fatal opioid overdoses, with no association with nonfatal overdoses. Abandoned house remediation was found to significantly reduce fatal and nonfatal opioid overdoses and should be considered alongside other essential treatment and harm reduction interventions for SUD. Neighborhood environmental interventions impact a growing list of health and safety outcomes and therefore should be attractive to policymakers and community residents alike seeking to address upstream drivers of health. Additional studies are warranted to explore mechanisms linking these interventions with overdose outcomes.

## Notes

Supplement 1. Trial Protocol and Statistical Analysis Plan

Supplement 2. eMethods. eTable 1. Secondary analysis of opioid overdose and acute opioid intoxication eTable 2. Secondary analysis of opioid overdose, acute opioid intoxication, and acute non-opioid intoxication eTable 3. COVID-19 sensitivity analysis eTable 4. Displacement analysis of opioid overdose

Supplement 3. Data Sharing Statement
