# Supplementary material for: Neighborhood Environmental Interventions and Opioid Overdose Rates
Source: JAMA Netw Open. 2026 Jul 31;9(7):e2626634. doi: 10.1001/jamanetworkopen.2026.26634 (PMC13428286; doi:10.1001/jamanetworkopen.2026.26634)
Supplement: Supplement 1. — Trial Protocol and Statistical Analysis Plan [file jamanetwopen-e2626634-s001.pdf]

Social and Behavioral Sciences Human Research Protocol *2-6-2022*

PRINCIPAL INVESTIGATORS: Zachary Meisel, MD MS, Eugenia C. South, MD MS

PROTOCOL TITLE: Studying the PhilAdelphia Resilience Project as a Response to Overdose (SPARROw)

1. INTRODUCTION AND PURPOSE:

1.1. Objectives: The objective of this proposal is to evaluate the effectiveness and implementation of two City of Philadelphia Resilience Project programs to reduce opioid overdose: (1) the Alternative Response 2 (AR2) that accompanies ambulances responding to overdoses and aims to deliver harm reduction and care linkage to people who refuse hospital transport, and (2) blight remediation of abandoned buildings and land. This application leverages a natural experiment within the Resilience Project implementation to evaluate its impact on overdose outcomes.

We propose to address the following Specific Aims:

SA1: Determine the effectiveness of the Resilience Project AR2 on opioid overdose outcomes for EMS patients who refuse hospital transport after overdose. Using a quasi-experimental design, we will compare the <u>proximal overdose outcomes</u> (rates and timing of treatment linkage) and <u>distal overdose outcomes</u> (non-fatal overdoses within 60 days and overdose deaths within 24 months) for patients who receive AR2 engagement compared to those who, due to timing or location restrictions, receive traditional EMS care alone.

SA2: Evaluate the impact of Resilience Project blight remediation activities on fatal and non-fatal opioid overdose. Blight remediation activities vary across time and space in Kensington. We will use a difference-in-difference design to compare fatal and non-fatal overdoses in the 60 days before and after any given remediation effort compared to the same time period in two nearby locations that did not get remediated but had comparable pre-blight remediation overdose rates. We will select one control location from within Kensington and one just outside the Resilience Project geographic boundary.

SA3: Evaluate implementation of the Resilience Project by a) characterizing the fidelity of the AR2 intervention in Kensington, b) measuring factors that moderate AR2 fidelity, including blight, c) assessing barriers and facilitators to AR2 implementation, and d) determining the costs for AR2 and blight remediation interventions. We will characterize AR2 fidelity by measuring which components are completed at each engagement (take-home naloxone, rapid treatment referral, transportation to treatment, family engagement, phone number acquisition, and/or phone follow-up). We will measure potential moderating factors including nearby blight at AR2 engagement locations and AR2 team composition. We will assess barriers and

facilitators to AR2 fidelity with interviews. Last, we will determine the costs for AR2 and blight remediation to inform sustainability and dissemination.

1.2. Background:

The opioid crisis has hit Philadelphia particularly hard, with over 46 overdose deaths per 100,000 residents in 2017, twice the national average. In response, the City of Philadelphia developed an overarching prevention strategy, the Resilience Project, which includes both services targeted toward people who use opioids and neighborhood environment interventions that address the blighted conditions where most overdoses occur. The Resilience Project focuses on Kensington, the neighborhood with the city's highest overdose rate and high rates of poverty, homelessness, and dilapidated neighborhood conditions.[2,3]

Experiencing an overdose often brings people who use opioids into contact with healthcare providers and represents an opportunity to improve outcomes through harm reduction and care linkage. For example, many people are brought to Emergency Departments after overdose and are linked to care through "warm hand off" programs.[4] However, among the 15,000 annual emergency medical services (EMS) calls in Philadelphia for overdose, up to 28% of patients refuse transport to the hospital and are, thus, at acute risk of later overdose. The Resilience Project AR2 aims to close this gap by engaging people who use opioids at the *time and place* of their overdose. The AR2 is a mobile medical unit – staffed with a social worker and paramedic – that accompanies ambulances responding to overdose calls in Kensington to engage in treatment or distribute naloxone to patients who refuse hospital transport.

Another Resilience Project program is blight remediation. The places where people live, work, and play are significant drivers of health.[5] This is particularly important in the opioid epidemic, as blighted neighborhood conditions are associated with drug sales, substance use, and morbidity.[6–16] Resilience Project blight remediation efforts include abatement of vacant lots and cleaning and sealing of abandoned houses. Changing the neighborhood context of high-risk overdose *places* such as Kensington could be an innovative method to augment opioid overdose prevention efforts, including the AR2.

This application leverages a natural experiment within the implementation of the Resilience Project to evaluate its impact on fatal and nonfatal opioid overdose. We hypothesize that interventions targeted at the *time and place* of opioid overdose – including the AR2 and blight remediation efforts – will be more effective at preventing overdose compared to standard EMS care or absence of environmental modifications.

2. CHARACTERISTICS OF THE STUDY POPULATION:

2.1. Target population and Accrual: Our target population are all people who have an opioid overdose in the Kensington neighborhood of Philadelphia, PA and surrounding areas. We are not enrolling people directly. Rather, we will be analyzing data from the

City of Philadelphia for people who meet our criteria. There are two primary ways in which a person may be part of our study – if they interact with the Alternative Response Unit (AR2) or traditional EMS care after an overdose. We will also look at all overdose deaths in this neighborhood. Of note, the intent of this project is to study opioid overdoses in Kensington; however, due to the nature of drug overdoses and the fact that many people use multiple substances, we recognize that often times it will be difficult or impossible to determine if the overdose was due to opioids. Therefore, we will be including encounters for all overdoses in our study.

2.2. Key Inclusion Criteria:

Inclusion criteria for the intervention group include all AR2 encounters where:

- The encounter is for a presumed overdose
- Patients are at least 18 years of age
- Located within the Kensington area (AR2 dispatch zone)
- From May 1, 2019 to April 30, 2021 (24 months)

Inclusion criteria for the comparator group include all EMS encounters where:

- The encounter is for a presumed overdose
- Patients are at least 18 years of age
- Located within the Kensington area (AR2 dispatch zone) and areas that are immediately adjacent
- From May 1, 2019 to April 30, 2021 (24 months)

2.3. Key Exclusion Criteria:

Exclusion criteria for the intervention group:

- Encounters for children younger than 18
- Encounters subsequently deemed non-overdose related
- Encounters where the patient is pronounced dead at scene
- Repeat encounter for a patient previously encountered during the study period

Exclusion criteria for the comparator group:

- EMS encounters for children younger than 18
- EMS encounters subsequently deemed non-overdose related
- EMS encounters where the patient is pronounced dead at scene
- Repeat EMS encounter for a patient previously encountered during the study period

Additionally, another part of the study will involve interviewing the AR2 team. We define the AR2 team as any individual involved in the creation and management of AR2, including direct AR2 staff members, leadership, and AR2 adjacent staff such as EMS workers who may interact with the AR2– we will aim to interview all such individuals (n = 40 people).

2.4. Subject Recruitment and Screening: For people who interact with the AR2 or traditional EMS, we will not be doing any specific recruitment to the study. The

population will be added to our study retrospectively and followed using city administrative data. Sources of research material consist primarily of administrative data already collected by the City of Philadelphia including information about all AR2 encounters, EMS data regarding non-fatal opioid overdose dispatches, Medical Examiner data regarding fata opioid overdoses, and Department of Behavioral Health data regarding substance abuse treatment. We will collect data over a 2-year time period.

We will recruit AR2 team members to participate in semi-structured qualitative interviews through talking with them directly about the study. We will not be recruiting AR2 team members via flyers or posters. We will rely on a snowball sampling technique, where AR2 team members that we speak with directly are able to refer to us other individuals they believe would be eligible to be interviewed.

2.5. Early Withdrawal of Subjects: N/A

2.6. Vulnerable Populations: N/A

2.7. Populations vulnerable to undue influence or coercion: N/A

3. STUDY DESIGN:

3.1. Design:

<u>AIM 1:</u> Determine the effectiveness of the Resilience Project AR2 on opioid overdose outcomes for EMS patients who refuse hospital transport after overdose.

a. **Rationale and theoretical framework:** Commonly, in Philadelphia and elsewhere, people who refuse transport to an ED after their overdose are at particular risk for repeat overdose and death as they typically do not receive, at the time of the event, referral to treatment, counseling, or take-home naloxone that could be obtained through "warm handoff" programs at the hospital.[28] This gap in prevention and treatment may contribute to significant levels of morbidity and mortality. The AR2 targets this unique and high-risk group of opioid users at the time and place of their overdose. Our evaluation of the AR2 will be guided by the Quality Framework for Emergency Treatment of Opioid Use Disorder as outlined by Samuels and colleagues.[25] In this framework, emergency care harm reduction interventions and treatment can be implemented and evaluated through a Donabedian structure-process-outcome perspective to assess a prehospital emergency approach to opioid use.[66] In Figure 7 we adapt this framework to the AR2 in which high quality prehospital care has the structural capacity to provide take-home naloxone and referral to evidence based treatment programs with immediate availability, harm reduction and treatment referral processes are delivered with high fidelity, and people who overdose are linked to care and subsequently are less likely to experience non-fatal and fatal overdose outcomes.

b. **Study design:** This quasi-experimental evaluation of the effectiveness of the AR2 will compare rates and time to treatment linkage, non-fatal overdoses within 60 days, and deaths within 24 months for people in the Kensington neighborhood who overdose, refuse EMS transport, and receive AR2 services with those who refuse EMS transport but for whom the AR2 is not dispatched.

c. **Intervention**: The AR2 is a neighborhood-based intervention launched by three City of Philadelphia public agencies (PDPH, PFD, MDO), supported by the City of Philadelphia and a multi-year SAMHSA implementation grant (RFA SP-17-005), which does not provide capacity for rigorous scientific evaluation. Please note the Penn team is not directly involved in implementing or running the AR2. The AR2 brings a specialized response unit, staffed by a paramedic lieutenant, a social worker, a case manager and a certified recovery specialist, to the scene for 9-1-1 calls that indicate overdose in the Kensington neighborhood. Each AR2 dispatch includes a paramedic and various combinations of other support staff. The AR2 accompanies – but do not replace – conventional ambulances dispatched for these calls. The ARU is dispatched between 10 AM and 6 PM, 7 days/week for all presumed overdose EMS calls in Kensington. While many overdoses occur during the day, many also occur at night. Daytime staffing of the ARU was initiated for logistical and budgetary purposes with the plan to extend hours if deemed effective. If the ARU arrives to the patient before the ambulance, the ARU team initiates medical treatment, including administration of naloxone.

The ARU delivers six core services on scene to patients who refuse transport to the hospital: 1) dispensing of take-home naloxone, 2) referral to a treatment facility that has immediate availability and can provide MAT, 3) transportation to the facility, 4) family engagement, 5) acquisition of patient telephone information for follow-up, and 6) connect over the phone with the patient in follow-up. If the ARU vehicle arrives after the ambulance, ARU staff provide backup support focused on harm reduction and linkage to care as needed. ARU interventions can occur whether or not naloxone has been administered. These services are, in other settings, limited to hospital-based and fixed community-based sites and are largely outside of the scope of traditional EMS care. While EMS has the capacity to provide take-home doses of naloxone (depending on availability), anecdotally uptake has been low in Philadelphia. Moreover, traditional EMS does not have the capacity to refer patients to treatment programs, provide transportation directly to treatment programs, or follow up with people who have experienced overdose. These patients, many of who are revived by naloxone, are at particularly high risk of repeat overdose and death and do not typically have access to harm reduction or OUD treatment in locations where they live and/or use drugs. Based on total EMS encounters for overdose in Philadelphia -- including those that receive naloxone and those that don't -- and our knowledge of EMS transport refusal rates in Kensington (approximately 25%-30%), we estimate between 300 and 500 ARU engagements per year, or 600-1000 engagements over our two-year study period.

d. **Comparator Group:** We will use EMS encounters for drug overdose that do not include AR2 arrival on-scene as a comparator group. There are multiple reasons for an EMS-only patient encounter for overdose, e.g., the 1) call is for an overdose outside of the geographic AR2 dispatch zone; 2) EMS dispatch occurs during an time in which the AR2 is not active (6PM-10AM); the 3) AR2 is not dispatched because an overdose is not presumed based on the dispatcher assessment; the 4) AR2 is unavailable because it is on-scene at another call; or 5) the ambulance and/or patient leaves before the AR2 arrives on-scene. The reasons for a patient to receive EMS-alone are largely determined by timing and location of the overdose as opposed to endogenous patient or provider-level characteristics. We will construct multiple comparison encounters based on these five reasons. Specific decisions will depend on the frequency we observe in our data collection. Ideally, encounters that are non-AR2 because of a competing simultaneous call represent an ideal comparator since they match the AR2 call in terms of time, date, and location. These simultaneous encounters will represent the primary comparators. As a secondary comparator group, we will also construct comparison encounters near a temporal or spatial AR2/non-AR2 boundary. Such discontinuities are also useful for identifying causal effects through a difference or difference-in-difference analysis.

<u>AIM 2:</u> Evaluate the impact of Resilience Project blight remediation activities on fatal and non-fatal opioid overdose.

a. **Rationale:** The blight remediation efforts taking place in Kensington as part of the Resilience Project may have an immediate impact on drug use behavior, which could result in fewer overdoses. For example, remediating abandoned buildings reduces the number of semi-private but easily accessible physical spaces in which drugs could being used. Abating vacant lots could affect the experience of stress in the neighborhood, which could result is less drug use.
b. **Study site and design:** This is a community level difference-in-differences study of the impact of blight remediation on opioid overdose outcomes taking place in the Kensington neighborhood (same location as Aim 1).
c. **Intervention:** The blight remediation activities included in this proposal are abandoned house remediation and vacant lot abatement. We will obtain the date, time, and exact geographic location of all blight remediation activities from the City of Philadelphia. At the start of the study, we will receive a list of activities to date, followed by a monthly report of all activities in the previous 30 days. Abandoned house remediation involves cleaning and properly sealing abandoned houses with secure plywood (clean and seal). This intervention is performed by the Department of License and Inspection. To date, 95 houses have received this intervention. In addition, these houses are also eligible to get a “doors and windows” intervention, which in involves painting doors and windows on the plywood, which gives the appearance that the house in lived in. This intervention is performed by the Community Life Improvement Program (CLIP). To date, 64 houses have received this intervention. See Figure 8 (previous page) for two before/after examples. Finally,

vacant lot abatement involves clearing trash from the lot. A select number of lots will also receive a clean and green intervention, similar to the intervention studied by our team, which involves grass seeding, erecting a low wooden post-and-rail fence, and monthly maintenance.

d. **Comparator Group:** For each blight remediation activity, we will randomly select two comparator locations that are at least 0.25 miles away that were eligible for but did not receive blight remediation in the 30 days prior to the intervention activity. The first comparator location will be in the Kensington zip code (comparator 1), allowing for a comparison in the neighborhood in which all Resilience Project activities are taking place. This will also allow us to evaluate if blight remediation can be linked to an absolute decrease in overdose without simply pushing drug use and overdose to a different location. The second comparator location will be outside the Kensington neighborhood in one of the immediately adjacent zip codes (comparator 2). This comparator allows us to evaluate the impact of blight remediation activities in a location where no Resilience Project efforts are taking place in order to account for any co-linear associations that may be due to other resilience project interventions but are independent of blight remediation.

We will use the ArcGIS tool Similarity Search to randomly pick the two comparator locations with unremediated abandoned houses or vacant lots for each blight remediation intervention location.[72] Using the tool, we will first find a block group either within Kensington (comparator 1) or immediately outside of Kensington (comparator 2) that has the same sociodemographic profile of the matched blight remediation area, but did not receive remediation. We will then determine the geographic center of each selected block group, which will serve as the point locations around which outcomes are measured.

<u>AIM 3:</u> Evaluate implementation of the Resilience Project by a) characterizing the fidelity of the AR2 intervention in Kensington, b) measuring factors that moderate AR2 fidelity, including blight, c) assessing barriers and facilitators to AR2 implementation, and d) determining the costs for AR2 and blight remediation interventions.

a. **Rationale and theoretical framework:** As with many interventions, the AR2 comprises a complex repertoire of behaviors in which staff must engage. The AR2 will deploy with different staff compositions and deliver different combinations of core components in each encounter. In order for the AR2 to be implemented as designed (fidelity), it is essential to understand which malleable factors – including neighborhood blight – modify its implementation. Assessing costs and resource use of both the AR2 and blight remediation is also critical to inform future efforts to implement, disseminate, and spread these interventions.

The measurement and analyses for Aim 3 are guided by the Consolidated Framework for Implementation Research (CFIR).[73] CFIR identifies intervention characteristics, factors outside the organization (outer setting), factors inside the organization (inner setting), provider characteristics, and processes that may affect implementation. Figure 9 shows the CFIR framework adapted for the proposed study. We will

measure use of six aspects of the AR2 for which it was designed to perform (fidelity). We will also characterize inner and out setting factors, individual provider characteristics and aspects of the AR2-patient engagement that may affect this implementation outcome. For example, the presence of a social worker (inner setting) at the time of engagement may increase the probability of treatment referral. Knowledge, self-efficacy and beliefs about the AR2 by its providers (individual) could impact how they engage with people who overdose. The presence of street blight (outer setting) could add stress, fear, and a sense of chaos to the AR2 encounter, affecting both the AR2 team and the overdose victim, limiting engagement.

b. **Characterize which AR2 components are completed (fidelity).** First, we will use the established AR2 database to characterize which AR2 dispatches result in: 1) dispensation of take-home naloxone, 2) referral to a treatment facility that has immediate availability and can provide MAT, 3) transportation to a treatment facility, 4) family engagement, 5) collection of patient telephone information, and 6) telephone follow up within 7 days of encounter. Last, we will characterize which AR2 dispatches lead to any interaction with the individual who experienced the overdose (as some persons leave the scene after being administered naloxone by police, EMS, or a bystander). AR2 staff record each of these variables in real-time for each AR2 dispatch. Data are imported into a secure database and stored at Philadelphia Fire Department headquarters.

c. **Measure factors that moderate AR2 fidelity, including blight.** We will characterize the extent to which select inner and outer setting factors as well as individual and process characteristics are associated with 1) any AR2 patient engagement, 2) overall fidelity, and 3) individual fidelity components. The outer setting characteristics we will explore are neighborhood environmental characteristics measured through direct observation within 72 hours of each AR2 engagement for engagements that occur after IRB approval of the study. The most relevant and malleable inner setting factor is staffing combination (e.g. presence or absence of social worker, CRS, and/or caseworker for each AR2 encounter).

Neighborhood environmental characteristics (outer setting) will be measured as follows. Each morning, we will obtain a list of the exact geographic location of all prior day's AR2 engagements from the AR2 team; similar to past work from our team, a trained study field team will then visit those locations that afternoon to take a full set of photographs,[65] which will be uploaded and stitched into 360° high-resolution panoramas using Microsoft Photosynth software. (Figure 10, previous page). Photographs will be taken within 72 hours of the AR2 engagement and we anticipate minimal change in environment during that period. Trained coders will code all photographs for level of blight using a code book developed by our team for prior work.[65] We will focus on visible elements of the neighborhood environment that we expect to be modifiable based on the Resilience Project blight remediation activities including trash, building conditions, vacant lots, and graffiti. Each photograph will be coded by two coders and discrepancies resolved by consensus.

Our primary interest is in studying the correlation between our blight measure and AR2 fidelity. We will regress fidelity on our measure of blight, testing whether high levels of blight are associated with lower program fidelity. We expect the AR2 to have 300-500 engagements per year. The Kensington zip code has 2,160 intersections, approximately the total number of unique outdoor locations. However, we do not expect that these intersections are equally likely to have overdoses. We simulated 366 overdoses occurring at these intersections, such that some intersections could have 10 overdoses, while most intersections have 0. This unequal chance of an overdose at each intersection does not induce clustering, but it does limit the observed variation in blight measures. We simulated in such a way that the most blighted intersections are the ones to have the most overdoses. Then we considered a test of correlation (or equivalently, a linear regression) between our blight measure and our fidelity measures. We have sufficient power to detect correlations more negative than -0.10.

Staffing combination (inner setting) is self-recorded by the AR2 providers. Due to varying AR2 staffing from shift to shift, we anticipate variability in dosage and fidelity by different crews, which will be unconfounded with encounter features, conditional on time and place. Our primary approach will be regression models, logistic regression for 0/1 outcomes and a proportional hazards model for time-to-event outcomes, in which our measures of dosage and fidelity are covariates. The power calculations for these analyses are identical to the Aim 1 power analysis for comparisons across time discontinuities. Rather than comparing across time periods, we will be comparing across high/low dose and high/low fidelity. Certain patients may be more receptive to AR2 services causing the AR2 to provide a greater dosage and maintain higher fidelity to the AR2 model. While we have no direct measure of "receptivity," we will use a collection of patient features potentially correlated with receptivity including patient substance abuse profile, number of prior overdoses, age, race, and sex. We will use propensity score weighting to reweight high dosage patients so their features collectively resemble the features of low dose patients. Outcome models using these weights will estimate the treatment effect of receiving a low dose, answering what would happen to low dose patients if instead they were give a high dose of AR2 services. Similarly, we can reweight patients receiving high fidelity AR2 response to resemble low fidelity AR2 response, so that the outcome model estimates the effect of receiving low fidelity for those patients who actually received low fidelity AR2. Although dosage and fidelity are not 0/1 measures, we will consider dichotomizing them if high/low dosage and high/low fidelity have clear boundaries in practice.

d. **Evaluating barriers and facilitators to AR2 implementation:** In order to supplement the approach to implementation analysis described above, we aim to add a qualitative approach to understanding and describing provider and process level individual perspectives that may influence implementation. This additional approach acknowledges that, while guided by theoretical implementation frameworks, a purely quantitative approach could miss important contextual aspects of implementation that

weren't captured in the quantitative measures. We will conduct individual semi-structured interviews with the AR2 team (n=40). Informed by the Theory of Planned Behavior, we will assess the range of perceptions related to norms, attitudes, beliefs, knowledge, and perceived behavioral control focused on patient engagement and delivery of AR2 services.[75] The interview guide will be framed around assessing "which parts of the AR2 seem to work and which do not" and how the team plans and reflects on processes for improving implementation. Interviews will be conducted and audio-recorded via Zoom by staff trained in qualitative interviewing. Transcripts will be entered into NVivo 12.0 (QSR, Doncaster, Australia) for analysis. Analysis will be overseen by Dr. Meisel who has extensive experience in conducting and analyzing qualitative interviews.[52,53,60,76,77] Using modified grounded theory, members of the team will discuss the transcripts and create a codebook.[78] Two study staff will pilot and then fully and separately code the transcripts. Analytic memos will be created for all nodes, describing key themes and a comprehensive list of relevant quotations from interviewees. Using the constant comparison methods, in which newly collected data will be compared with categories that emerge from previously collected data, all memos will be analyzed for consistency and disagreement. We will also examine differences in concepts for interviewees by their profession (e.g., social worker or paramedic).

e. **Measure program costs:** We will determine the cost of the AR2 and the blight remediation interventions from the perspective of the City of Philadelphia, including its Department of Public Health and Fire Department. This portion of the project will be conducted with guidance from colleagues at CHERISH, the NIDA-funded multi-institutional substance use treatment health economics center, for which Dr. Meisel (mPI) leads the Policy and Dissemination Core. Our approach will follow standard micro-costing methods.[79] Micro-costing is a technique in which all inputs consumed in an intervention are identified and quantified in detail; then these resources are converted into pecuniary terms to produce a cost estimate.[80] It is most appropriate when there are no alternative data sources because an intervention is novel and services are not always reimbursed. Resources will include personnel time, travel costs, and materials directly used by the AR2 and blight remediation programs, and these resources will be valued based on salaries, benefits, and other unit costs provided by the relevant City of Philadelphia departments. To facilitate national comparisons, we will also value personnel costs at standard wage and fringe benefit rates for relevant personnel categories published by the U.S. Bureau of Labor Statistics.[81] Supervision and other overhead costs will be estimated based on interviews with City of Philadelphia staff members. The results of this analysis will inform future decisions regarding sustaining implementation and expansion of these novel place-based interventions for delivering harm reduction and care linkage services for a uniquely vulnerable population of patients who overdose.

4. METHODS:

Methods to evaluate the Alternative Response Unit (AR2):
Quasi-experimental studies showing relationship between urban blight remediation and overdoses.
We will evaluate the impact of blight remediation on fatal and nonfatal opioid overdoses using quasi-experimental methods. Across the AR2 dispatch zone, various vacant lots that have been cleaned and greened will serve as our treatment group. Randomly selected control lots will be matched to these treated lots. We will perform regression analyses to understand associations between blight remediation and overdoses.

Methods to evaluate the impact of blight remediation on fatal and nonfatal opioid overdoses:
Using 360° photographs to measure level of blight in the neighborhood environment.
360° photographs will be taken of the immediate surrounding outdoor environment where AR2 responses occur and where the control participants were at that same time. Photographs will be coded and analyzed. We will use this photographic technique to evaluate the level of blight around AR2 engagements.

4.1. Study Instruments: We will develop an interview guide for interviews which will include, when relevant: the perception of the AR2, their experience taking care of overdose patients, and challenges they face while working as part of the AR2, and their suggestions for improvement.

4.2. Group Modifications: There will be no group modifications.

4.3. Method for Assigning Subjects to Groups: N/A

4.4. Administration of Surveys and/or Process:
Process for Review of Databases: We will use data from four different City of Philadelphia departments: Philadelphia Department of Public Health (PDPH), Philadelphia Fire Department (PFD), Department of Behavioral Health and Intellectual disAbilities, and Managing Director's Office (MDO). These data are not currently linked between departments (we have letters of support from each agency). AR2: the AR2 dispatch and encounter data will be shared from both the PDPH and PFD databases. These data include 1) date, time, and location for all AR2 interactions, 2) demographic information about each participant including name and date of birth, and home address (when available), and 3) information about which components of the AR2 intervention were delivered during each engagement and in follow up.

Data related to linkage to care outcomes will be extracted from the DBHIDS Enterprise Data Warehouse. This data system includes all Medicaid plus uninsured claims for substance abuse treatment and care in Philadelphia. There is robust Medicaid penetration among opioid users in Philadelphia. In 2017, there were over 15,000 unique beneficiaries with opioid use disorder using behavioral health services, up from 9,000 in 2010.[67] DBHIDS has committed to sharing these data in a manner that can be linked to EMS and

AR2 visit. This ensures that we will be able to track the substantial number of people who use opioids and who remain in Philadelphia through the behavioral health system after they have come into contact with the AR2 or EMS.

Data regarding fatal opioid overdose will come from the PDPH through the Philadelphia Office of the Medical Examiner, which sits within the PDPH. These data will include the name, date of birth, and death location for all fatal opioid overdose victims. The second distal outcome is non-fatal overdose, data which will come from both the PFD and the PDPH. The PFD will provide data for all EMS overdose related encounters, including date, time, and location of the dispatch; patient name and date of birth; patient home address (when available); and outcome of encounter (refused transport vs. transport to a hospital).

4.5. Data Management:

Data that originates in the City of Philadelphia will stay within the jurisdiction of the City of Philadelphia. One person, acting as a shared Data Analyst between the Penn study team and the City of Philadelphia, will have access to City data. This individual will be funded using SPARROw grant dollars and have joint non-paid appointments in City departments providing de-identified data to the PIs for analysis. With the appointments in City departments, the individual will function as an intern within the City departments with access to department data.We will have a data sharing agreement that allows the provision of de-identified data to the PIs. Working as an intern in each data contributing department, the Data Analyst will be responsible for preparing the dataset, including data transformation of variables needed for the statistical modelwithin the city setting. Only de-identified information will be sent from the City of Philadelphia to the PIs. Interview data will be recorded and transcribed. Transcripts will only have a subject ID and not be specifically labeled by name. Interview data and photographs data will be managed by the PIs, Eugenia South and Zachary Meisel. Existing datasets will be transferred via a secure, electronic file transfer to computer server space dedicated to the proposed study and protected with firewall and encryption technologies managed by the IT Department of the University of Pennsylvania Perelman School Of Medicine. Drs. South and Meisel will have master access to this drive and all data will only be accessible through a secured log-in. Abby Dolan is the project manager. Kehinde Oyekanmi is the research coordinator. Other co-investigators, research coordinators, research assistants, and data analysts will only be granted access at the discretion of Dr. South and Dr. Meisel.

4.6. Subject Follow-Up: N/A

5. STUDY PROCEDURES:

5.1. Detailed Description: Please see study design section for complete description of ARM and EMS opioid related care. The study is not involved in executing these city services, rather we are studying the outcomes using city administrative data.

5.2. Data Collection: Please see study process section above. For interviews, we will find a convenient time for each individual interested in participating in the interview. Interviews will not take place during work hours unless approved by the individual's supervisor. Interviews will take place in a private location so that no one can hear the interview other than study staff. Interviews will be recorded and transcribed. Study staff will send each participant their own interview transcript, allowing the participant to review the transcript and provide comments or corrections . For photographs, the study team will get a list each morning of the locations of AR2 engagements over the prior 24 hours. Two members of the study team will then take a 360 photograph at the location of the engagement. Full, 360° sets of photographs will be taken from the street corner closest to each case (corresponding to the overdose location) and control location using a standardized field protocol. Photographs will be uploaded and stitched into 360° high-resolution panoramas using Microsoft Photosynth software. Trained coders blinded to case/control status will code all panorama photographs for 60 visible elements of the built and social environments using a detailed codebook. Photographs will be assessed by multiple coders and any discrepancies will be resolved by consensus. These photos will exclude people when at all possible. If people are inadvertently in the photograph, no effort will be made to identify them. The photos will be kept confidential and destroyed at the end of the study. No facial photographs will be published.

5.3. Genetic testing: N/A

5.4. Use of deception: This study does not include the use of deception.

5.5. Statistical Analysis:

<u>**AIM 1 Analysis:**</u>

a. **Analysis plan and power calculation.** The unit of analysis is the patient-encounter. While our primary analysis will only include a patient's first encounter during the study period, we will consider analyses of patients with multiple encounters over the study period, possibly switching from comparison to intervention group, while taking care to avoid survivor bias. We will follow each patient from first encounter until either a subsequent overdose or death, whichever comes first. The following plan includes three designs - a paired encounter analysis, a comparison across time periods, and a difference-in-difference analysis. Our preferred approach to minimize the risk of confounding is the paired encounter analysis, but for some outcomes the anticipated number of paired encounters would provide insufficient power. We can address this limitation in statistical power by comparing outcomes across different

time periods when the AR2 is operating and when it is not, with a slightly greater risk of confounding, but likely to have greater power. A difference-in-difference analysis is our final fallback position.

b. **Paired encounter analysis.** For the analysis of paired encounters (one AR2 and a non-AR2 occurring while the AR2 was on another call), we will use an exact McNemar's test for paired data for 0/1 outcomes (death, relapse) and a Cox proportional hazards model for paired data for censored time to event outcomes (time to treatment linkage, time to subsequent overdose).[68] For each AR2 encounter we try to identify a non-AR2 encounter at the same time period within the AR2 response zone. If we find one, these pair of encounters are labeled as a pair and we will follow the patients for 60 days for linkage and non-fatal overdose, and 24 months for fatal overdose, and record outcomes. If multiple non-AR2 encounters happen simultaneously, then conditional logistic regression and stratified Cox models will extend the analysis from a 1-to-1 pairing to a 1-to-many matching. For our power calculations we assume a 1-to-1 pairing, a one-sided test for a reduction in adverse outcomes with $\alpha = 0.05$ and a power level of at least 0.80. To detect a 50% reduction in the death rate attributable to the AR2 (assumed reduction from 10% to 5%), we need 370 pairs of encounters. To detect a 50% reduction in the rate of repeat non-fatal overdoses (from 30% to 15%), we need 105 pairs of encounters.[69] To detect a 50% reduction in the hazard rate of time to subsequent overdose (equivalent to roughly 19% having a subsequent overdose within one year to 9.5%), we need 200 pairs of encounters. To detect an increase of 50% in the odds of seeking treatment within 60 days of an overdose (from 26% seeking drug treatment after an overdose within 60 days to 35% with AR2), we need 360 pairs of encounters.[70]

c. **Comparisons across time discontinuities**. For outcomes underpowered with a paired analysis, we will compare AR2 encounters during AR2 operational hours (10AM-6PM) to non-AR2 encounters outside of AR2's operational hours (6PM-10AM) within the Kensington zip code. Since comparison encounters will not be occurring at the same times as the AR2 encounters, there is a risk of confounding. We will first assess whether AR2 and non-AR2 patients encountered differ on likely confounders (e.g. age, race, sex, substance use profile such as type of drug, injected or not). Ideally focusing exclusively on patient encounters closest to AR2 time boundaries (near 10AM and 6PM) will provide sufficient observations and minimize the risk of confounding. If we find no differences (e.g. patients overdosing earlier in the day resemble those who overdose later in the day) then we will analyze the data as a simple binomial test of proportions (for 0/1 outcomes) or estimate the hazard ratio (for time to event outcomes). If we do find differences, then we will propensity score reweight comparison cases on age, sex, race, and substance use profile so that they do match.71 The following power calculation assumes no propensity score reweighting, but if propensity score reweighting is needed, the effective sample size, computed as $(\sum w_i)^2/\sum w_i^2$ where w_i is the propensity score weight, will need to equal the needed sample size in the power analysis. This depends on how many comparison encounters closely resemble the AR2 patients. To detect a 50% reduction in the death rate

attributable to the AR2 (assumed reduction from 10% to 5%), we need 380 encounters in each arm. To detect a 50% reduction in the rate of repeat overdoses (from 30% to 15%), we need 105 encounters in each arm. To detect a 50% reduction in the hazard rate of time to subsequent overdose (equivalent to roughly 19% having a subsequent overdose within one year to 9.5%), we need 200 encounters in each arm. To detect an increase of 50% in the odds of seeking treatment within 60 days of an overdose we need 380 encounters in each arm.

d. **Difference-in-difference**. To further protect against confounding, we will compare encounters across times and places simultaneously. If within zip code 19134 those who overdose earlier in the day differ from those who overdose later in the day, but those differences are also reflected in the population outside zip code 19134, then a difference-in-difference analysis can use encounters outside 19134 to learn the expected difference between patients with early overdoses and later overdoses and appropriately adjust the within 19134 estimated treatment effect. For example, let $\hat{p}_{01} - \hat{p}_{00}$ represent the estimated difference in the rate of seeking treatment for those outside 19134 during AR2 operating hours ($\hat{p}_{01}$) and the rate of seeking treatment for those outside 19134 and outside of AR2 operating hours ($\hat{p}_{01}$). Since the AR2 never operates outside of 19134 this difference captures the expected difference treatment seeking for those who overdose at different time periods. Then the difference-in-difference estimate $((\hat{p}_{11} - \hat{p}_{10}) - (\hat{p}_{01} - \hat{p}_{00}))$ adjusts the treatment contrast within 19134 ($\hat{p}_{11} - \hat{p}_{10}$) by subtracting off the observed difference by time of day for an untreated sample ($\hat{p}_{01} - \hat{p}_{00}$). In practice, we compute ratios of odds ratios for 0/1 outcomes and ratios of hazard ratios for time-to-event outcomes.

Because difference-in-difference estimates require estimation of three comparison quantities, the estimates are less precise and have greater sample size demands. We compute the number of encounters needed in each area (within 19134 and outside but near 19134) and time period (AR2 operational hours and AR2 non-operational hours). To detect a 50% reduction in the death rate attributable to the AR2 (assumed reduction from 10% to 5%), we need 600 encounters in each area in each time period. To detect a 50% reduction in the rate of repeat overdoses (from 30% to 15%), we need 170 encounters in each area and time period. To detect a 50% reduction in the hazard rate of time to subsequent overdose, we need 320 encounters in each area and time period. To detect an increase of 50% in the odds of seeking treatment within 60 days of an overdose we need 700 encounters in each area and time period.

AIM 2 Analysis:

a. **Analysis plan and power calculation.** We will analyze separately abandoned home remediation and vacant lot abatement, but both will have a similar analysis structure. We will use a conditional Poisson regression model of the form $\log(E(\text{count})) = \beta_0 + \beta_1\text{clean} + \beta_2\text{post} + \beta_3\text{clean} \times \text{post}$, where clean is a 0/1 indicator of whether the location went through blight remediation and post is a 0/1 indicator of whether the time period measured is post-blight remediation in the treated location. The conditional Poisson model accounts for the matched pairs of location. $\exp(\beta_3)$ is of

primary interest, a ratio of rate-ratios, contrasting the relative change in overdose rates in the treated area pre- versus post-remediation with the relative change in overdose rates in the comparison locations.
We anticipate at least 55 blight remediated locations with which we can pair with 55 un-remediated locations. The average buffer area will have 1.2 overdoses per month. Under such circumstances we will be able to detect reductions of 50% or more in overdose rates with power 0.80.

AIM 3 Analysis

a. **Evaluating barriers and facilitators to AR2 implementation.** In order to supplement the approach to implementation analysis described above, we aim to add a qualitative approach to understanding and describing provider and process level individual perspectives that may influence implementation. This additional approach acknowledges that, while guided by theoretical implementation frameworks, a purely quantitative approach could miss important contextual aspects of implementation that weren't captured in the quantitative measures. We will conduct individual semi-structured interviews with the AR2 team (n=40). Informed by the Theory of Planned Behavior, we will assess the range of perceptions related to norms, attitudes, beliefs, knowledge, and perceived behavioral control focused on patient engagement and delivery of AR2 services.[75] The interview guide will be framed around assessing "which parts of the AR2 seem to work and which do not" and how the team plans and reflects on processes for improving implementation. Interviews will be conducted and audio-recorded via Zoom by staff trained in qualitative interviewing. Transcripts will be entered into NVivo 12.0 (QSR, Doncaster, Australia) for analysis. Analysis will be overseen by Dr. Meisel who has extensive experience in conducting and analyzing qualitative interviews. Using modified grounded theory, members of the team will discuss the transcripts and create a codebook. Two study staff will pilot and then fully and separately code the transcripts. Analytic memos will be created for all nodes, describing key themes and a comprehensive list of relevant quotations from interviewees. Using the constant comparison methods, in which newly collected data will be compared with categories that emerge from previously collected data, all memos will be analyzed for consistency and disagreement. We will also examine differences in concepts for interviewees by their profession (e.g., social worker or paramedic).

6. RISK/BENEFIT ASSESSMENT:

6.1. Risks: The primary risk is loss of confidentiality through a data breach – although all data will be kept at the City of Philadelphia. The risk is small however, given the data safety plan in place and the experience of the research team conducting community-based studies. There is also a small psychological risk to the AR2 team during interviews, as

they will be asked to describe how the AR2 is doing and reflect on challenging situations. Interview participants will be told that they are able to choose not to answer questions that are upsetting to them. They will also be encouraged to reach out to a study team member who is trained to help them work through the questions or provide them with resources if needed. A final risk involves our research team who will go into the field to take photographs. The team will always go into the field in teams of two. Their safety is of the utmost importance. Field teams will go out during daytime hours at times when many people will be outside. If the AR2 team deems that a site of engagement felt unsafe, they will tell our team and we will forego that photograph. The team will also be trained to be aware of their surroundings and to leave the scene if they feel unsafe at any point.

6.2. Benefits: Participants will not directly benefit from being included in the study, as we are studying interventions that are already in place by the City of Philadelphia. The AR2 team will benefit from participation in interviews as they may feel good in contributing to knowledge generation relevant to the opioid epidemic and their perspectives will ideally be used to improve implementation and dissemination of their work. This research will have significant impact on our ability to prevent fatal and non-fatal opioid overdose. The experience of the opioid crisis in Philadelphia in general and Kensington specifically is valuable to cities across the country. Lessons learned about the AR2 and blight remediation from this proposal will be directly improve state and local health departments ability to implement and improve interventions focused on preventing opioid related death. Given the magnitude of the opioid epidemic, and the innovation of the interventions we are studying, this proposal has the potential for large benefit.

6.3. Risk-benefit assessment: Based on our careful assessment of the risks versus benefits, and our previous work, we judge the risks to be reasonable in relation to the benefits.

6.4. Subject Privacy: We are not directly interacting with any opioid overdose patients. For AR2 staff, we will make sure that interviews are completed in a private location away from other AR2 or other fire department staff.

6.5. Subject Confidentiality: All data and records generated throughout the course of the study will be kept confidential in alignment with University of Pennsylvania Institutional Policies. Only authorized study personnel will have access to the study data and records for the purposes of conducting the study. If results of the study are published or presented at scientific meetings, no names or other identifying information will be included.

Individual transcripts of AR2 team interviews will not be shared with the City of Philadelphia to ensure the AR2 team feels free to discuss facilitators and barriers without retaliation. We will only share results with the City of Philadelphia after results have been analyzed for high level themes and insights, and properly de-identified.

Data will be managed by the mPIs, the City of Philadelphia, and the study team. Data will be transferred via a firewall-protected, secure, electronic file transfer to computer server space dedicated to the proposed study and protected with firewall and encryption technologies. Only the mPIs and our city Partners will have master access to the linked data. Co-investigators, research assistants, and other staff will be granted data access at the discretion of the mPIs and access will be documented.

How will confidentiality of data be maintained? Check all that apply.

☐ Paper-based records will be kept in a secure location and only be accessible to personnel involved in the study.

☒ Computer-based files will only be made available to personnel involved in the study through the use of access privileges and passwords.

☐ Prior to access to any study-related information, personnel will be required to sign statements agreeing to protect the security and confidentiality of identifiable information.

☒ Whenever feasible, identifiers will be removed from study-related information.

☐ A Certificate of Confidentiality will be obtained, because the research could place the subject at risk of criminal or civil liability or cause damage to the subject's financial standing, employability, or liability.

☒ A waiver of documentation of consent is being requested, because the only link between the subject and the study would be the consent document and the primary risk is a breach of confidentiality. (This is not an option for FDA-regulated research.)

☐ Precautions are in place to ensure the data is secure by using passwords and encryption, because the research involves web-based surveys.

☒ Audio and/or video recordings will be transcribed and then destroyed to eliminate audible identification of subjects.

☐ Other (specify):

6.6. Protected Health Information:

The Penn study team will only receive de-identified datasets from the City of Philadelphia. For AR2 team members who are interviewed, the following information will be collected:

1. Name
2. Street address, City, Zip code, Latitude and Longitude
3. All elements of dates (except year) for dates directly related to an individual and all ages over 89
4. Date of birth
5. Sex
6. Ethnicity

6.7. Compensation: City of Philadelphia employees will not be compensated for their participation due to City regulations. Compensation will be made to those AR2 team members we interview who are not City of Philadelphia employees. Participants will be compensated $20 upon interview completion. Participants will receive a gift card.

Participants will not be required to provide their social security number to be compensated. For City of Philadelphia employees who are not able to be compensated for their participation, the study team will use food delivery apps (such as Door Dash, Uber Eats, etc) to send them refreshments equaling no more than $20.

6.8. Data and Safety Monitoring: A Data Safety Monitoring Plan (DSMP), will be instituted to maintain data integrity and confidentiality. Data will be transferred via a firewall-protected, secure, electronic file-transfer to computer server space dedicated to the proposed study both within the City of Philadelphia and the University of Pennsylvania, and protected with firewall and encryption technologies. The City of Philadelphia maintains access to all city related data, and only authorized study personnel will be able to access this data. For data that is collected directly by the University of Pennsylvania, only the mPIs (Dr. Meisel and Dr. South) will have master access to this server and will be required to authenticate themselves via password each time the data are accessed.

Protected health information (PHI) will be collected by the AR2 and used to link people across multiple city data sets. This PHI data will be necessary to the conduct of the proposed study and include: name; date of birth, telephone numbers, and linked claims data. The Penn study team will only receive de-identified datasets from the City of Philadelphia. No PHI will be disclosed to anyone who is outside of the proposed research team. We will institute strict precautions and security procedures to maintain data integrity and confidentiality:

(1) All data collected by University of Pennsylvania will be stored on a centralized computer server at the Perelman School of Medicine at the University of Pennsylvania. This server will be dedicated to the proposed study and will be protected with up-to-date "fire-wall" electronic security technology.
(2) Only Drs. Meisel and South (mPI), will have master access to this server and the data.
(3) Computer administrators, co-investigators, project coordinators, and other staff will only be granted data access at the discretion of the Principal Investigators. Furthermore, this access will only be granted as needed for finite periods of time throughout the study.

The mPIs and the project manager will be responsible for providing and documenting appropriate user access to the study database, and preventing against major sources of data security problems: unauthorized internal access to data, external access to data, and malicious intent to destroy data and systems. This user access will ensure that only appropriate and authorized personnel are able to view, access, and modify trial data. Modifications to data will be performed in a manner that documents data modification, user access associated with modification, data associated with modification, and values prior to modification. The mPIs and the project manager will also be responsible for optimizing database performance, reliability and backup of data. External, unauthorized access to data is prevented through cooperative efforts of the mPIs, the project manager, and network and systems administrators. Highly successful measures will be employed

through network firewall technologies to prevent unauthorized external access to data repositories.

Drs. Meisel and South will perform an interim analysis at the halfway point of the study to determine the effect to date of the AR2 and blight remediation on opioid overdose. These results will be shared with our City of Philadelphia partners to inform ongoing prevention efforts.

Drs. Meisel and South (mPI) will be directly responsible for identifying and reporting all serious adverse events, protocol deviations/violations, and unanticipated events to the IRBs and funding agency promptly, as appropriate.

7. INFORMED CONSENT:

7.1. Consent Process: The research team will not have direct contact with any individuals who overdose, rather we will track their outcomes via administrative data. Thus, we will apply for a waiver of informed consent. We will consent AR2 team to be part of the study.

7.2. Waiver of Informed Consent:

We request a waiver of consent for this study, based on the following:

- The research involves no more than minimal risk to the subjects.
- The waiver of consent will not adversely affect the rights and welfare of the subjects.
- The research could not practicably be carried out without the waiver. Due to the time period covered and the procedures being investigated, patients may not have an ongoing relationship with the health system and many may be lost to follow-up.
- The research is unlikely to generate additional pertinent information which should be communicated to subjects after participation.

8. RESOURCES NECESSARY FOR HUMAN RESEARCH PROTECTION:

Our team (please also see Investigator Biosketches), based at the Penn Injury Science Center, a CDC Injury Control Research Center that focuses on time and place-based approaches to injury science and the Center for Emergency Care Policy and Research, is specifically experienced to investigate the Resilience Project from geospatial, emergency care structure-process-outcomes, and implementation science perspectives. We also partner with the Columbia University ICRC. The team brings together complementary and successful research experiences that demonstrate our ability to implement this study. We have a long history of publications and grants (including multiple active and completed R01 and center grants) focusing on substance use disorder

treatment and prevention, violent injury, and urban health, including RCTs studying provider-patient interactions around opioid prescriptions and use, and RCTs and quasi-experimental studies testing the health and safety effects of place-based change. The mPIs and Co-Is have expertise and a long history of collaboration in the areas of prescription opioid use and overdose (Meisel, Perrone); EMS (Meisel); implementation science and behavioral health (Meisel, Mandell); and placed-based public health interventions (South, Wiebe, Branas, MacDonald). The research team includes experts in geospatial analysis (Wiebe, Tam, Branas); quasi-experimental analysis (Ridgeway, Branas, MacDonald); and public health community outreach, dissemination science, and knowledge translation (Meisel, Solomon).

Our research team will also include Abby Dolan, MPH, Project Manager; and Kehinde Oyekanmi, BS, Clinical Research Coordinator. Ms. Dolan has experience working on studies in opioid prescriptions and opioid overdose including a RCT. Ms. Oyekanmi has managed studies that examine the risks and protective factors in urban neighborhood environments for stress, crime, violence, and health.

We have long standing established relationships with our municipal and community partners who we partner with to implement this proposal. We have partnered with several departments within the City of Philadelphia government to create this proposal (see letters of support). Our primary partnership is with the Philadelphia Department of Public Health (PDPH). PDPH is charged with securing, analyzing, and reporting trends in opioid use, including both fatal and nonfatal overdoses; use of addiction treatment services; opioid prescribing practices; and medical consequences of drug use. As such, the PDPH leads the design and implementation of the Resilience Project, including the AR2. All work is done in close collaboration with the Medical Examiner's Office (MEO), which is a division of the PDPH and investigates and conducts comprehensive toxicology testing on all fatal overdose victims. Our PDPH partners include Kendra Viner, PhD, MPH, Opioid Surveillance Program Manager; Jeffrey Hom, MD MS, Policy Advisor; and Raynard Washington, PhD, Chief Epidemiologist. We have a long-standing relationship with these partners - Dr. Perrone has worked with Dr. Viner over the last year creating a real-time response to spikes in overdose (published in the NEJM), and Dr. Meisel and Dr. Perrone have worked with both Dr. Viner and Dr. Hom while serving on the Mayor of Philadelphia's Opioid Task Force subcommittees.[50] Please note, the PDPH is preparing an application for the CDC's Overdose Data to Action Funding Opportunity (RFA-CE-19-1904).

We also partner with the Philadelphia Fire Department (PFD) to evaluate the AR2 and obtain EMS-related data. Crawford Mechem, MD, the PFD EMS Medical Director, is a faculty member in the Penn Department of Emergency Medicine with whom Drs. Meisel, South, and Perrone have worked for many years. Crystal Yates is the PFD Assistant Deputy Commissioner and Paramedic Chief. We also partner with the Department of Behavioral Health and Intellectual disAbilities Services (DBHIDS) to obtain data related to linkage to substance abuse treatment through Medicaid and uninsured claims data. Our partners include Geoffrey Neimark, MD, Chief Medical Officer for Community Behavioral Health; Andrea Brooks, Special Coordinator to the Commissioner for Strategic Priorities; and Barbara Bunkle, PhD, Director of Enterprise Data Services. Dr. Mandell (Co-I) has a long-standing collaborative relationship with Dr. Neimark

and DBHIDS, having partnered on multiple prior studies. Finally, the Resilience Project is coordinated by the Managing Director's Office (MDO), and we partner with Angelina Ruffin, MS, Director of Performance Management, to coordinate data acquisition and analysis across multiple city agencies.

The SPARROw Steering Committee will be made up of the mPIs (Meisel, South), the PISC director (Wiebe), the study Project Manager, and one representative from each city agency involved (Viner, Mechem, Brooks, Ruffin). The Steering Committee will meet monthly in person to review study progress, ensure smooth data collection and linkage, and guarantee open lines of communication between all study partners.

In addition, we will integrate community organizations and members through the Resilience Project's existing framework for community engagement. The MDO, in which the Resilience Project sits, has partnered with community members and elected officials to hold monthly community meetings in Kensington. "The El Barrio es Nuestro" (The Neighborhood is Ours) meetings are held in both English and Spanish. In addition, the Resilience Project Community Advisory Committee meets bi-weekly and includes representatives from community groups in Kensington including New Kensington Community Development Corporation, Impact Services, and Esperanza Health. We will meet on a quarterly basis with the Community Advisory Committee and community members to inform this study, help with interpretation of results and dissemination of results to the community.
