# Supplementary material for: Neighborhood Environmental Interventions and Opioid Overdose Rates
Source: JAMA Netw Open. 2026 Jul 31;9(7):e2626634. doi: 10.1001/jamanetworkopen.2026.26634 (PMC13428286; doi:10.1001/jamanetworkopen.2026.26634)
Supplement: Supplement 2. — eMethods. eTable 1. Secondary analysis of opioid overdose and acute opioid intoxication eTable 2. Secondary analysis of opioid overdose, acute opioid intoxication, and acute non-opioid intoxication eTable 3. COVID-19 sensitivity analysis eTable 4. Displacement analysis of opioid overdose [file jamanetwopen-e2626634-s002.pdf]

Supplemental Online Content

South EC, Seeburger E, Dolan A, et al. Neighborhood environmental interventions and opioid overdose rates. *JAMA Netw Open*. 2026;9(7):e2626634.
doi:10.1001/jamanetworkopen.2026.26634

eMethods.

eTable 1. Secondary analysis of opioid overdose and acute opioid intoxication

eTable 2. Secondary analysis of opioid overdose, acute opioid intoxication, and acute non-opioid intoxication

eTable 3. COVID-19 sensitivity analysis

eTable 4. Displacement analysis of opioid overdose

eReferences

This supplemental material has been provided by the authors to give readers additional information about their work.

© 2026 South EC et al. *JAMA Network Open*.

eMethods

Nonfatal overdose determination methods

Data for EMS encounters was acquired from the Philadelphia Fire Department's TripTix electronic patient care report (ePCR) database. Encounters had to occur within the study geography. If the ePCR record did not contain a latitude and longitude point as determined by the computer-aided dispatch system, the recorded street address for the encounter was geocoded using the Decentralized Geomarker Assessment for Multi-Site Studies (DeGAUSS). Encounters without latitude and longitude coordinates and incomplete addresses were excluded. EMS encounters cancelled by dispatch or police on-scene, as well as encounters where the patient was deceased on arrival, were also excluded. To determine if an EMS encounter was a nonfatal overdose, the responding paramedic must have indicated in the ePCR that they administered naloxone as part of their care. Administration of naloxone is a protocolized action; paramedics are trained to look for cyanosis, pinpoint pupils, agonal respirations, and lack of responsiveness to painful stimuli (e.g., sternum rub) as indicators of respiratory arrest consistent with an opioid overdose. We considered these trained assessments by licensed medical professionals as reliable confirmations of overdose. A narrative chart review of 100 random encounters where naloxone was administered found 95 (95%) of these encounters were true opioid overdoses.

Additional robustness check: Permutation test

We conducted 1,000 permutations for the effect of all environmental interventions on nonfatal and fatal overdose as our test, randomly generating distributions by week. Compared to the distribution of results from our randomly generated models, the coefficient of interest in our main analysis models for nonfatal and fatal overdoses was statistically significant (both p's <0.001), meaning that the observed relationships between the environmental interventions and overdose outcomes are unlikely to be by chance.

Additional robustness check: Acute intoxication of opioids and non-opioids

Our secondary analyses sought to capture opioid overdoses in concert with acute opioid intoxication that did not meet the threshold for overdose and/or acute intoxication of other substances (e.g., synthetic cannabinoids, phencyclidine). To capture acute opioid intoxication (Supplemental Table #1), EMS encounters where naloxone was not administered but the paramedic indicated their primary impression was opioid use were added to the existing dataset of naloxone patients. Primary impression is determined by a variety of on-scene factors, including patient symptoms, information provided by the dispatcher, patient, and bystanders, and the presence of drug paraphernalia. To additionally capture acute intoxication involving other substances (Supplemental Table #2), EMS encounters where the responding paramedic classified the run as an overdose/poisoning (as determined by the "found to be" value in the ePCR), but where the primary impression was not opioid use and naloxone was not administered, were added to the existing dataset of naloxone and opioid use patients. With these two secondary analyses, we sought to capture the intervention's effect on the entire landscape of substance use in the neighborhood.

Sensitivity analysis: COVID-19 Pandemic

The COVID-19 pandemic was associated with worsening opioid overdose outcomes. The pandemic also impacted city services in our study ZIP code.[34] To account for potential impact on our results, we

© 2026 South EC et al. *JAMA Network Open*.

examined if there was a differential treatment effect pre- and post- COVID related shutdown, defined as the week of March 15, 2020, when the City of Philadelphia announced its stay-at-home order. We measured this by including an interaction term in our analysis that differentiated before and after this date in the regression model and found that the post-COVID time period did not significantly interact with the effect of the neighborhood environmental interventions on nonfatal overdoses (see supplemental table #3). There were too few fatal overdose observations separately between the pre- and post-COVID time periods to conduct an analysis with the interaction term.

© 2026 South EC et al. *JAMA Network Open*.

**Supplemental Table 1. Impact of neighborhood environment interventions on fatal and nonfatal opioid overdoses and nonfatal acute opioid intoxication in study ZIP code, Philadelphia, 2019-2021**				
	Any intervention (all interventions combined)	Community trash cleanup	Abandoned house remediation	Vacant lot cleanup
Fatal overdoses %, (95% CI)	-6.6 (-10.5, -2.4)***	1.3 (-5.1, 8.2)	-17.6 (-26.2, -7.9)***	-4.9 (-12.5, 3.3)
Nonfatal overdoses %, (95% CI)	-1.4 (-4.1, 1.4)	-1.3 (-5.0, 2.5)	-16.3 (-22.1, -10.0)***	11.4 (5.0, 18.2)***
***denotes p<0.001				

© 2026 South EC et al. *JAMA Network Open*.

Supplemental Table 2. Impact of neighborhood environment interventions on fatal and nonfatal opioid overdoses, nonfatal opioid intoxication, and nonfatal acute non-opioid intoxication in study ZIP code, Philadelphia, 2019-2021				
	Any intervention (all interventions combined)	Community trash cleanup	Abandoned house remediation	Vacant lot cleanup
Fatal overdoses %, (95% CI)	-6.6 (-10.5, -2.4)***	1.3 (-5.1, 8.2)	-17.6 (-26.2, -7.9)***	-4.9 (-12.5, 3.3)
Nonfatal overdoses %, (95% CI)	-2.1 (-4.4, 0.3)*	-1.4 (-4.6, 1.9)	-14.0 (-19.1, -8.5)***	7.1 (1.7, 12.6)**
*denotes p=0.05<br>**denotes p=0.01<br>***denotes p<0.001				

© 2026 South EC et al. *JAMA Network Open*.

Supplemental Table 3: COVID-19 sensitivity analysis, nonfatal overdoses<br>COVID-19 Cutoff: Study week 63 (March 15th, 2020 shutdown) before/after			
	**Coefficient of interest, expressed as percent**	**COVID-19 Interaction term coefficient, expressed as percent**	**Percentage change in expected value**
All neighborhood environment interventions	-1.0%	1.0%	2.0%
Community trash cleanup	-2.3%	1.2%	3.4%
Vacant house remediation	-6.1%	-6.6%	0.6%
Vacant lot cleanup	-3.1%	8.9%	11.1%

© 2026 South EC et al. *JAMA Network Open*.

**Supplemental Table 4.** Displacement Analysis of Fatal and Nonfatal Opioid Overdoses to Non-Intervention Blocks in study ZIP code, 2019-2021				
	Any intervention (all interventions combined)	Community trash cleanup	Abandoned house remediation	Vacant lot cleanup
Fatal overdoses %, (95% CI)	1.1 (-1.8, 4.1)	2.6 (-0.9, 6.3)	2.7 (-2.9, 8.6)	-3.1 (-8.9, 3.1)
Nonfatal overdoses %, (95% CI)	-0.9 (-3.5, 1.7)	-1.3 (-4.3, 1.8)	5.9 (-0.5, 12.6)	-4.7 (-10.0, 0.9)

© 2026 South EC et al. *JAMA Network Open*.
