# Supplementary material for: Neighborhood Environmental Interventions and Opioid Overdose Rates
Source: JAMA Netw Open. 2026 Jul 31;9(7):e2626634. doi: 10.1001/jamanetworkopen.2026.26634 (PMC13428286; doi:10.1001/jamanetworkopen.2026.26634)
Supplement: Supplement 3. — Data Sharing Statement [file jamanetwopen-e2626634-s003.pdf]

Data Sharing Statement

South. Neighborhood Environmental Interventions and Opioid Overdose Rates. *JAMA Netw Open*. Published July 31, 2026. doi:10.1001/jamanetworkopen.2026.26634

Data
Data available: No

Additional Information
Explanation for why data not available: Data owned by City of Philadelphia
